# Supplementation of Diet With Different n-3/n-6 PUFA Ratios Ameliorates Autistic Behavior, Reduces Serotonin, and Improves Intestinal Barrier Impairments in a Valproic Acid Rat Model of Autism

**DOI:** 10.3389/fpsyt.2020.552345

**Published:** 2020-09-09

**Authors:** Jinpeng Wang, Baihong Zheng, Dan Zhou, Jie Xing, Honghua Li, Jiayu Li, Zehui Zhang, Beilin Zhang, Ping Li

**Affiliations:** ^1^ Department of Cardiology, The Second Hospital of Jilin University, Changchun, China; ^2^ Department of Pediatrics, The Second Hospital of Jilin University, Changchun, China; ^3^ Department of Developmental Pediatrics, The Second Hospital of Jilin University, Changchun, China; ^4^ Department of Developmental and Behavioral Pediatrics, The First Hospital of Jilin University, Changchun, China; ^5^ Department of Physiology, College of Basic Medical Sciences, Jilin University, Changchun, China

**Keywords:** polyunsaturated fatty acids, autism spectrum disorder, valproic acid, intestinal barrier, serotonin

## Abstract

The implication of different dietary n-3/n-6 polyunsaturated fatty acids (PUFAs) ratios has been investigated in some neurodevelopmental disorders (including autism and depression). However, the mechanisms underlying the effects of different PUFAs ratios on the autism are still poorly understood. In the present study, a valproic acid (VPA) rat model of autism was used to study the effects of diet with different n-3/n-6 PUFA ratios on the autism, and the underlying mechanisms explored. Our results showed that rats with prenatal administration of VPA took less response time to sniff three odorants in the olfactory habituation/dishabituation tests, had lower frequency of pinning and following patterns, and had decreased hippocampal 5-hydroxytryptamine (5-HT), increased serum 5-HT and downregulated expression of tight junction protein (occludin and claudin-1) in the colon. However, supplementation of n-3/n-6 PUFAs (1:5) in the VPA treated rats ameliorated the autistic behaviors, increased hippocampal 5-HT and tight junction expression in the colon, and decreased serum 5-HT. In conclusion, dietary supplementation of n-3/n-6 PUFAs (1:5) significantly improves VPA-induced autism-like behaviors in rats, which may be, at least partially, related to the increased hippocampal 5-HT. Furthermore, this diet can increase the expression of tight junction proteins to improve intestinal barrier impairment.

## Introduction

The autism spectrum disorder (ASD), a type of developmental neurobiological disorders, is characterized by the pervasive impairments of social interaction and communication capabilities, and the restricted, repetitive, and stereotyped behaviors, activities, and interests (1). Generally, the prevalence of autism is about 1% in young and adult population (2). It is has been revealed that both environmental and genetic factors are related to the pathogenesis of ASDs (3).

Clinically, valproic acid (VPA) is used as an anticonvulsant and mood-stabilizing drug, and there is evidence showing that prenatal VPA exposure may cause maladaptive behaviors and birth defects and increase the risk of autism (4). It has been reported that epileptic mothers with VPA treatment during the pregnancy have autistic offspring (5). In our study, results showed rats treated with VPA at embryo day 12.5 (E12.5) developed ASD syndromes. Prenatal administration of VPA (Pre-VPA) in rats is an acceptable practice in the establishment of animal model of autism, and these autistic rodents have a wide range of characteristics similar to those in humans (6).

It has been reported that gastrointestinal symptoms (such as abdominal pain, diarrhea, vomiting, chronic constipation, and gastroesophageal reflux) are common in ASD children, but the prevalence of these symptoms varies in ASD children of different studies. As compared to healthy children, ASD children are more susceptible to gastrointestinal symptoms (7). The intestinal tract is also referred as “second brain” due to its abundance of enteric nerves, because it has vital roles and functions in the immune system as well as neurological system. Increasing evidence shows that signals of the intestinal immune disturbances can be transduced to the brain through multiply pathways, influencing the emotions and behaviors (8). Normal intestinal barrier function plays a vital role in maintaining immunity and overall wellbeing (9). At least, over 50 proteins have been identified to play significant roles in the regulation of tight junction in the mucosal endothelial layer and the intestinal permeability (10). The tight junction complexes include four families of transmembrane proteins. They are tricellulin, occludin, junctional adhesion molecules, and claudins. Normally, the tight junction proteins maintain the polarization of intestinal barrier to regulate the paracellular passage to transport small molecules only, such as leukocytes, ions, solutes and water.

Some studies, including ours, have shown that prenatal administration of VPA in rats may cause some cerebral and intestinal abnormalities similar to those in ASD patients (11–13).

Dietary supplementation of n-3 PUFAs can improve the intestinal barrier function in rats. It has been reported that eicosapentaenoic acid (EPA) and docosahexaenoic acid (DHA) can prevent deoxynivalenol-induced intestinal cell injury and reinforce barrier function, which is associated with the inhibition of necroptosis signaling pathway (14). Furthermore, Zhu et al. found that marine oil supplementation was beneficial for the improvement of intestinal barrier function and inhibition of CRH/CRHR1 signaling pathway and mast cell density (15). Thus, we speculate that dietary supplementation of n-3/n-6 PUFAs may be conducive to improve the impaired intestinal barrier in autistic rats.

Dietary supplementation of n-3 PUFAs has been studied in the treatment of neurologic disorders such as ASD. n-3/n-6 PUFAs are abundant in the central nervous system (CNS), particularly DHA (n-3) and to a lesser extent arachidonic acid (AA, n-6) (16). PUFAs cannot be produced in humans and should be obtained *via* diet (17). However, the intake of n-3 PUFAs in the diet has declined. In Western diet, n-6/n-3 PUFAs ratio is up to 16:1, which is significantly higher than 1:1 in the traditional diet. This is of special concern because competition between dietary PUFAs from plant sources and long-chain PUFAs for the same enzymes functioning for elongation and desaturation. Therefore, excess n-6 PUFAs may lead to the translocation of n-3 PUFAs in cellular membranes. n-6 and n-3 PUFAs are derived from eicosaoids generated by EPA and AA, respectively, and may exert almost opposite effects. Thus, the balance between n-3 and n-6 PUFAs is important for the homeostasis. It has been found that the imbalance between n-3 and n-6 PUFAs may cause vasoconstriction (and subsequent restricted or decreased bloodstream), thrombosis, and inflammation (18). In children with attention-deficit hyperactivity disorder (ADHD) or ASD, the serum levels of AA, EPA, and DHA are low, while the ratio of n-6/n-3 PUFAs is higher, which is significantly associated with ASD symptoms (19). Therefore, supplementation of dietary n-3/n-6 PUFAs may affect the autistic behaviors.

In this study, rats received prenatal administration of VPA to induce ASD-relevant symptoms, following behavioral assessment, colonic pathology, and biochemistry. In addition, the effects of daily supplementation of n-3/n-6 PUFAs (1:5) on the autism were further investigated by behavioral tests and detections of serum and hippocampal 5-hydroxytryptamine (5-HT) and the expression of tight junction proteins in the colon. The present study was under-taken to investigate the potential of daily supplementation of n-3/n-6 PUFAs in prenatal VPA induced autism in rats.

## Materials and Methods

### Animal Model

The animal study was reviewed and approved by the Ethics Committee of the Second Hospital and College of Basic Medical Sciences of Jilin University, and all procedures were conducted according to the Guideline for Animal Care and Use of Jilin University. Five adult male Wistar rats (220–240 g) were selected to mate with 10 female ones (2 weeks old and 350–500 g). After mating, gestational day 1 (G1) was defined as the day of presence of sperms on a vaginal smear. The pregnant rats at E12.5 were grouped as follow: Control group (n = 3) (rats were intraperitoneally administered with 100 μl of 0.9% saline) and VPA group (n = 7) (rats were intraperitoneally administered with VPA at 600 mg/kg once).

On the 12th day after birth, 8 male young offspring of mothers in the control group served as controls. In addition, 24 male young offspring of mothers with VPA treatment were divided into VPA group, A group and B group (n = 8 per group).

### Diet

All neonatal rats received breast-feeding for 21 days. Then, these rats were given ad libitum access to standard chow containing 11.6% fat, 18.8% protein, and 66.7% carbohydrate in the control group and VPA group. In the “A” group, rats were given ad libitum access to n-3 free chow; in the “B” group, rats were given ad libitum access to chow supplemented with n-3/n-6 PUFAs at 1:5. Nutrients of each type of chow are as shown in **Table 1**.

**Table 1:** Diet macronutrient profiles.

Ingredients (g/kg)	Control	A (n-3 free)	B (n-3/n-6 = 1:5)
Casein	200	200	200
Corn starch	388	388	388
Sucrose	150	150	150
methionine	3	3	3
Sunflower seed oil	0	660	110
Vitamin mixture	10	10	10
Mineral mixture	35	35	35
Cellulose	47	47	47
Choline	2.5	2.5	2.5
Calcium	4	4	4
lard	0	300	300
Linseed oil	0	0	40
Fish oil	0	0	510

### Behavioral Tests

#### Olfactory Habituation/Dishabituation

The behavior test begins at the 11th week after birth. Olfaction is a major communicative way between rats (20). Rats were separately housed in clean plastic cages (24 cm × 14 cm × 13 cm). A detachable plexiglas covered with a 1.25-cm hole was used for behavioral habituation testing on the 30th day. After 2-min acclimation, a saline-saturated swab was placed through the hole to a 2.5-cm deep position and remained for 5 min with 3 cycles. Then, a swab saturated with another rat’s urine was used for habitation testing for 5 min with 3 cycles. Subsequently, a beer-soaked cotton swab was used for 5 min with 3 cycles. Before and after each test, 75% ethanol was used to sterile the plexiglas cover. A digital stopwatch was employed to record the response time to active sniffing. The mean response time was calculated.

#### Social Play Behaviors

The experiments were done in a sound attenuated but dimly-lit space. A Plexiglas cage (40 cm × 40 cm × 60 cm) was used as the testing arena. The cage floor covered with 2-cm-deep wood shavings. A zoom video equipped with DVD and LCD monitor was employed to monitor and record the behaviors of tested rats.

The rats aged 3 months were placed into the experimental apparatus individually and allowed to accommodate to the environment for 10 min, which repeated two days. On the day of testing, in order to promote their social interaction motivation and further boost the expression of social play behaviors during the testing, the individual rat stayed in a separated environment for 3 h till the testing (21). In each test, two rats from the same group were placed in the experimental apparatus and stayed for 15 min. The difference of body weight between two rats was no more than 10 g and these rats had no common social experience previously.

The following parameters were scored: (1) Following: moving forward to or tailing after the other experimental subject, who was running away; (2) Frequency of pinning: one rat lying on its back on the floor while the other rat standing over it. In the social play of rats, such posture is most characteristic one when one tested subject is under a playing plea solicited by its counterpart who rotates to tis dorsal surface.

### Detection of Hippocampal and Serum Serotonin Levels

After behavioral test, rats were anesthetized with chloroform and killed by decapitation, the brain and blood were collected, and hippocampus was carefully separated. The blood was centrifuged (1500 × g, 10 min), and the supernatant was collected for the detection of 5-HT level. In addition, 500 μl of ice-cold Tissue Protein Extraction solution (Beijing CoWin Biotech) was used to homogenize rat hippocampus (50 mg). The homogenates were centrifuged (10,000 × g, 20 min at 4°C). BCA Kit (Beijing CoWin Biotech) was employed to determine the total protein concentration. According to the manufacturer instructions, an ELISA kit (Tianjin Anoric Biotech, China) was employed to detect hippocampal and serum 5-HT level.

### Hematoxylin and Eosin (H&E) Staining

After behavioral assessment, animals were sacrificed, and the colons were harvested for further H&E staining. The colonic tissues were fixed in 10% buffered neutral formalin and then embedded in paraffin. Colon sections (5 μm) were obtained for routine HE staining with standard protocol. These sections were evaluated independently in a blinded way.

### Western Blotting

The colonic tissues were homogenized in lysis buffer for 30 min, followed by centrifugation. The supernatant was collected, and the protein concentration was determined with BCA method. Then, the supernatant containing 50 μg of protein was boiled for 5 min, and then subjected to sodium dodecyl sulfate polyacrylamide gel electrophoresis for protein separation. Then, the proteins were transferred onto nitrocellulose membranes which were then treated with 5% non-fat milk in TBST containing 10 mmol/L Tris-HCl (pH 7.5), 150 mmol/L NaCl, and 0.05% Tween-20 at room temperature to block nonspecific reactivity for 1 h. Following primary antibodies were used: occludin (1:1,000, ABclonal, no: A12621, Wuhan, China), claudin-1(1:1000, ABclonal, no: A2196, Wuhan, China) and anti-GAPDH (1:1,000, Boster Biotechnology, BM1985, Wuhan, China).

### Statistical Analysis

SPSS version 22.0 (Statistical Product and Service Solutions Inc., Chicago, IL) was used for statistical analysis. Data are expressed as mean ± standard error (SEM). One way analysis of variance (ANOVA), followed by least significant difference (LSD) test was employed for comparisons among groups. A value of P < 0.05 was considered statistically significant.

## Results

### Effects of Dietary n3/n6 PUFAs Supplementation on the Behaviors

**Figure 1:**
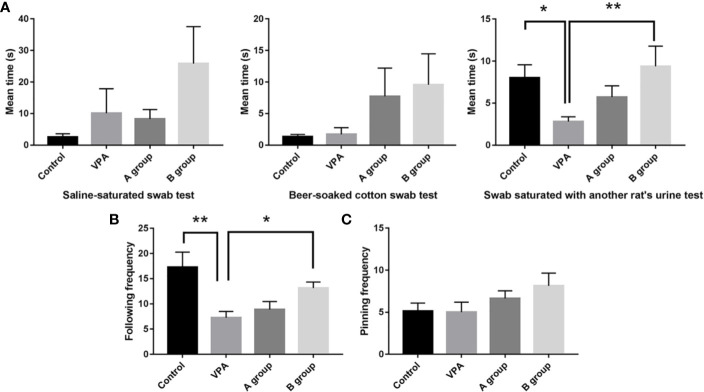
Effect of dietary n3/n6 PUFAs supplementation on the olfactory habituation/dishabituation and social play behavior. **(A)** Olfactory habituation/dishabituation; **(B)** Following frequency; **(C)** Pinning frequency. Data are expressed as mean ± SEM, n = 8. *P < 0.05 vs. VPA group. **P < 0.01 vs. VPA group.

Rats in the B group with n3/n6 = 1:5 supplementation were more sensitive to three odorants in the olfactory capability test (**Figure 1A**). Compared with control rats, the urine sniffing time reduced significantly in the VPA group (P < 0.01). In social play behavior testing, the following frequency in the B group was markedly higher than in the VPA group; as compared to the control group, the following frequency reduced markedly in the VPA group (**Figure 1B**). In the pinning frequency test, male offspring with prenatal VPA exposure had no difference with control ones (**Figure 1C**).

### Effects of Dietary n3/n6 PUFAs (1:5) Supplementation on the Hippocampal and Serum 5-HT Levels

**Figure 2:**
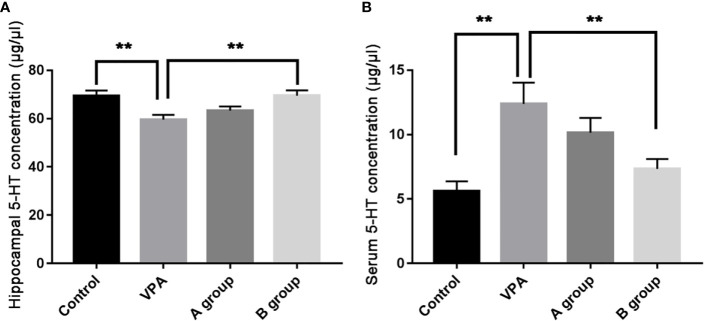
Hippocampal and serum 5-HT levels (μg/μl) in different groups. **(A)** Hippocampal 5-HT level; **(B)** serum 5-HT level. Data are expressed as means ± SEM. **P < 0.01: vs. VPA group. Control group, n = 7; VPA group, n = 6; A group, n = 5; B group, n = 5.

As shown in **Figure 2**, as compare to the control group, the hippocampal 5-HT level decreased significantly in the VPA group (P < 0.01, ANOVA). The hippocampal 5-HT level in the B group was markedly higher than in the ASD group (P < 0.01, ANOVA). Additionally, the serum 5-HT level in the VPA group was significantly higher than in the control group (P < 0.01, ANOVA) and the serum 5-HT level in the B group was markedly lower than in the VPA group (P < 0.01, ANOVA).

### Effects of Dietary n3/n6 (1:5) Supplementation on the Colonic Histology and Tight Junction Protein Expression

**Figure 3:**
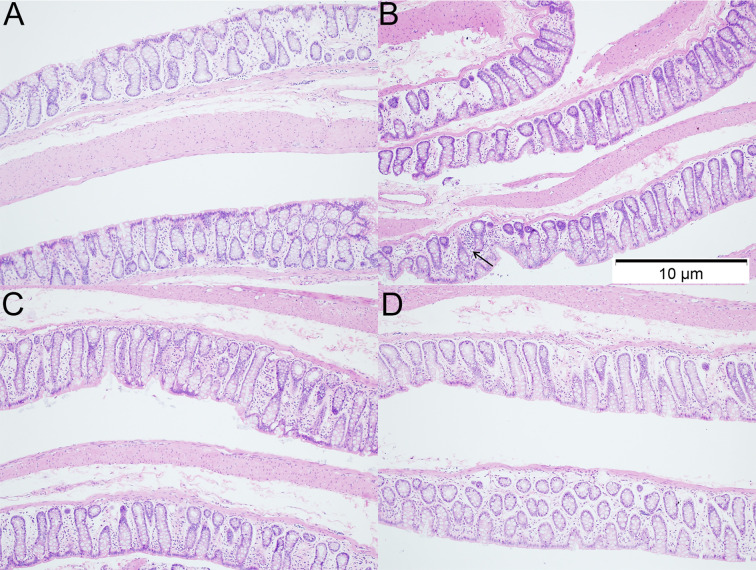
Histology of colonic tissues in different groups (HE staining; ×100). **(A)** control group; **(B)** VPA group; **(C)** A group; **(D)** B group. Arrows indicate more inflammatory cells were observed in the colon of rats with VPA treatment.

H&E staining of colon sections was employed to evaluate the histological changes in 12 weeks-old rats. As shown in H&E staining, more inflammatory cells were observed in the colon of rats with VPA treatment. However, the inflammatory was ameliorated after dietary n3/n6 PUFAs (1:5) supplementation (**Figure 3**).

**Figure 4:**
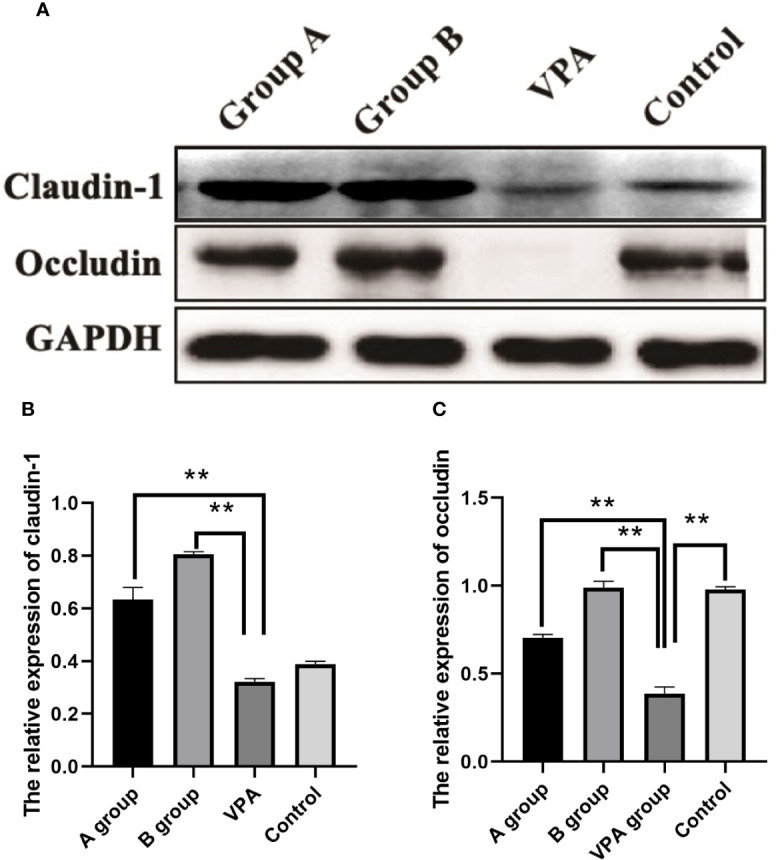
Expression of claudin-1 and occludin in the colonic tissues of different groups. Downregulated expression of occludin after VPA treatment. Upregulated expression of claudin-1 and occludin after dietary n3/n6 (1:5) supplementation. **(A)** Representative Western blot images of the protein bands. **(B)** Fold change of Claudin-1. **(C)** Fold change of occludin. Results are presented as mean ± SEM. Differences were analyzed using one-way ANOVA followed by LSD test. n = 3 in each group. **P < 0.01, compared with VPA group.

In the family of epithelial tight junction proteins, occludin and claudin-1 are important. As shown in Western blotting, the expression of occludin was significantly downregulated in the VPA group as compared to the control group. However, dietary n3/n6 PUFAs (1:5) supplementation significantly up-regulated the expression of occludin and claudin-1 in the VPA-treated rats (**Figure 4**).

## Discussion

Serotonin (or 5‐HT) is mainly found in the gastrointestinal tract (60%–90%), platelets and CNS of animals and humans, and it plays important roles in the regulation of intestinal movement, intestinal sensing and signaling, and secretion (22). *In vitro* studies have indicated that 5‐HT affects tight junction expression in Caco‐2 intestinal epithelial cells (23). 5-HT is involved in the modulation of hypotonicity-induced increase of duodenal mucosal permeability (24). Dong et al. found that the intestinal and plasma 5-HT levels were higher in the stressed animals, and the elevated 5-HT level could lead to diarrhea. 5-HT and diarrhea delayed the renewal of intestinal epithelial cells and impaired the mucosal barrier and absorption (25). In autism population and their relatives, the blood 5-HT level increases, implicating the involvement of 5-HT in the pathophysiology of autism (26). Therefore, the elevated serum 5-HT level in ASD patients may ameliorate the gastrointestinal dysfunction. Our results also showed the serum 5-HT level increased in the VPA-treated rats. However, after dietary supplementation of PUFAs, the serum 5-HT level in the VPA group decreased significantly, which may be associated with the improvement of intestinal barrier function.

H&E staining indicated the increased inflammatory cells and the loss of crypt structure in the colonic tissues of VPA group. However, after dietary supplementation of PUFAs in the VPA-treated rats, inflammatory cells in colonic tissues reduced, the structural impairment was lessened, the expression of junction proteins (occludin and claudin-1) in the colon was also upregulated significantly, which may be related to the decreased serum 5-HT level. In the *in vitro* model of enterocytes, 5-HT (0–2.5 mM) was able to regulate the expression of occludin in the basolateral cells in a dose dependent manner: exogenous 5-HT was able to decrease the occludin expression (23). This was consistent with our findings: the serum 5-HT level reduced after supplementation of PUFAs in the VPA group, which was accompanied by the elevated expression of occludin in the colon.

Compared with non-autistic population, the brain 5-HT concentration reduces significantly in the ASD population (27). Lower 5-HT level in the early brain development of rats may result in the neuroanatomical defects, such as abnormally tiny somatosensory barrels and dendritic arbors, fewer dendritic spines, and decreased synaptic density. In male young adults with ASD, a negative correlation has also been observed between low serotonergic neurotransmission and high blood 5-HT concentration, which is also referred as serotonin anomaly (28). According to the causal relationship between low serotonin and autism, rats lacking TPH2 gene (a protein coding gene and known to cause low 5-HT concentration in the brain) have abnormal serotonin synthesis in the brain and present autism-related symptoms, including the impairment of social communication and interaction capabilities, and disposition for restricted, repetitive behaviors and interests (29, 30). The behavioral complexity of ASD, characterized by deficits in learning, memory, emotion, and social functioning, suggests underlying alterations in multiple brain area (31). Hippocampus is an important brain region related to the etiology of ASD. It has been reported that decreased hippocampal neuronal size with increased cell packing density and the presence of less complex dendritic arborization are related to the disrupted neuronal maturation in ASD (32). Intriguingly, Narita et al. reported that prenatal VPA exposure increased hippocampal 5-HT level and pre-frontal DA level in rats (33). Our study also showed that hippocampal 5-HT level decreased and autism-related behaviors (olfactory habituation/dishabituation and social play behavior) were present in the VPA-treated rats, which may be associated with the decreased hippocampal 5-HT level. After dietary n-3/n-6 PUFAs (1:5) supplementation, the autistic behaviors in the VPA group were ameliorated and the hippocampal 5-HT level increased, suggesting that the behavioral improvement of autistic rats is associated with elevated hippocampal 5-HT level. n-3 PUFAs are crucial for the development and function of CNS. Increasing evidence from randomized placebo-controlled trials, animal experiments, and epidemiological studies has shown that n-3 PUFAs deficiency in the diet may lead to the development of affective mental disorders, and dietary supplementation with n-3 PUFAs may be helpful for the treatment of these disorders. It has been reported that n-3 PUFAs are better to increase the cell membrane fluidity as compared to n-6 PUFAs (34). It seems that patients with depressive disorder have decreased membrane fluidity due to the composition changes of PUFAs, which affects neurotransmitter binding, membrane enzymes and receptor activities (35, 36). It is reported a reversal diet with adequate n-6 and n-3 PUFA given during the lactating period to rats originating from alpha-linolenic acid-deficient dams was able to restore both the fatty acid composition of brain membranes and several parameters of the dopaminergic and serotonergic neurotransmission (37). Hereby, the membrane composition of n-3/n-6 PUFAs may exert significant influence on the monoaminergic systems, and the normalization of membrane structure by regulating n-3/n-6 PUFAs ratio may be employed to ameliorate ASD symptoms.

In the future, more studies are required to confirm the importance of n-3/n-6 PUFAs in the treatment of ASD.

## Data Availability Statement

The raw data supporting the conclusions of this article will be made available by the authors, without undue reservation.

## Ethics Statement

The animal study was reviewed and approved by the Ethics Committee of Second Hospital and College of Basic Medical Sciences of Jilin University.

## Author Contributions

JW designed the work, collected and analyzed data, and drafted the manuscript. JW, DZ, BHZ, JZ, HL, JL, ZZ, and BLZ collected and analyzed data and revised the manuscript; and PL contributed to the concept and design of this study, reviewed, and revised the manuscript.

## Funding

This study was supported by the Natural Science Foundation of China (No. 81602847), Youth Foundation of Health Commission of Jilin Province (No. 2015Q007), and Youth Foundation of Norman Bethune Medical College of Jilin University (No. 2015026021).

## Conflict of Interest

The authors declare that the research was conducted in the absence of any commercial or financial relationships that could be construed as a potential conflict of interest.
